# Sun and Shade

**Published:** 1892-03

**Authors:** Emma Cetlinski


### SUN AND SHADE.

For Hall's Journal of Health.

A little cloud will surely mar,
The sun's effulgence, dear;
And as we see it from afar,
We feel the darkness near.

And so, one little deed will cast,
A shadow o'er our life;
And thus the darkness holds us fast,
We live through pain, and strife.

But through it all still shines the sun
Behind the darkened sky,
And soon the gloomy clouds pass on;
Again the light is nigh.

God sends the light and darkness too,
He sends each pain and care;
But ah! God sends us joys anew,
And sunlight everywhere.

Oh! Mary, dear, when grief doth reign
In thine own troubled heart;
Look far beyond and comfort gain,
With but this simple thought.

Though gloomy are the clouds that dim,
Our horizon anon;
The sun will shed its beams again
And joy'll be thine, dear one.

—EMMA CETLINSKI.

